# Clinical relevance of seasonal changes in the prevalence of ureterolithiasis in the diagnosis of renal colic

**DOI:** 10.1007/s00240-016-0896-3

**Published:** 2016-06-17

**Authors:** Hiroki Fukuhara, Osamu Ichiyanagi, Hiroshi Kakizaki, Sei Naito, Norihiko Tsuchiya

**Affiliations:** 1 Department of Urology, Nihonkai General Hospital, 30 Akiho-cho, Sakata, Yamagata 998-8501 Japan; 2 Department of Urology, Yamagata University Faculty of Medicine, 2-2-2 Iida-nishi, Yamagata, Yamagata 998-9585 Japan

**Keywords:** Ureterolithiasis, Seasonal, Etiology, Emergency, Diagnosis

## Abstract

Ureterolithiasis is one of the most frequently diagnosed urologic diseases worldwide. Its annual incidence in Japan increased three-fold from 1965 to 2005. Ureterolithiasis incidence is affected by numerous factors, including race, sex, body weight, fluid intake, and climate. Here, we aimed to address the latter by considering the effect of seasonal variation on stone incidence and incorporating this information into a predictive model for differential diagnosis of ureteral stone from other conditions with similar presentations. We retrospectively identified 491 patients in our emergency department computer database who complained of back, flank, or lower abdominal pain during 2007–2015. Among them, 358 had stones, as confirmed by computerized tomography or plain abdominal X-ray of kidney–ureter–bladder. We also charted the mean ambient temperatures in our city for a year. The cases of ureteral stones paralleled the ambient temperatures, peaking during the hottest weather. Univariate analysis identified 13 factors associated with ureteral stones. Multivariate analysis narrowed the number to eight: age <60 years, male sex, short duration of pain (<6 h), nausea/vomiting, hydronephrosis, hematuria, history of urinary stone(s), and summer (July–September in Japan). Pain appearing during summer was nine times more likely to be due to a ureteral stone than was pain appearing during other seasons. We incorporated the eight variables identified into a predictive logistic regression model, which yielded good prediction of ureteral stones. Awareness that hot weather is associated with increased incidence of ureterolithiasis could facilitate differential diagnosis, and our prediction model could be useful for screening for ureterolithiasis.

## Introduction

Ureterolithiasis is one of the most prevalent urological diseases all over the world, including Japan [1–3]. The annual incidence of upper urinary tract stones in Japan has steadily increased, and in 2005, it was three times that in 1965 [2], which is similar to trends in many other countries [3–6]. The etiology of ureterolithiasis is considered to be multifactorial [7]. Race, sex, body weight, body mass index, diet, volume of fluid intake, geographic localization, and climate changes contribute to stone formation in the kidney [7].

Back, flank, and lower abdominal pain are some of the most important symptoms accompanying urinary stone disease; ureterolithiasis, however, does not involve specific symptoms [8–10]. Therefore, radiological imaging is often necessary to definitely differentiate ureterolithiasis from other conditions with similar symptoms at initial presentation [11]. The non-contrast computed tomography (NCCT) scan is valuable in clinical practice to detect ureteral stones with high sensitivity and specificity, regardless of the size, location, and chemical composition [10]. However, there are increasing concerns regarding radiation-induced cancers caused by diagnostic computed tomography (CT) [12, 13]. It is well known that ureterolithiasis is a recurrent disease, and when affected patients undergo CT scans repeatedly in their lifetime, they are at an increased risk of malignancy due to a potentially high cumulative effective dose of radiation exposure [12, 13]. Therefore, clinical X-ray exposure should be optimized to a minimum level of radiation without reducing the diagnostic relevance.

Clinical algorithms to predict the presence of ureteral stones have been produced based on the symptomatology of ureterolithiasis [9, 14]. To date, however, whether the season at presentation of symptoms contributes to diagnosis of ureteral stones remains unclear, although many reports have demonstrated seasonal variations in urinary stone incidence [15–17].

The aim of the present study was to examine the influence of seasonal variations in ureteral stone incidence upon the differential diagnosis of potential renal stones and to develop a prediction model, including a seasonal factor to aid in the diagnosis of patients complaining of flank, back, and lower abdominal pain.

## Patients and methods

### Participants, selection and exclusion criteria, and ethics statement

We retrospectively reviewed the computer database of medical records for patients aged ≥15 years who visited our emergency department (ED) between April 1, 2007 and March 31, 2015. The exclusion criteria were as follows: (1) absence of back, flank, or lower abdominal (including right or left lower quadrant) pain; (2) insufficient examination (lack of definitive radiological or urinalysis evidence or signs of hydronephrosis); (3) abnormal vital signs (subjective or objective fever or hypotension); (4) C-reactive protein (CRP) ≥ 6 mg/L in cases where leukocytes were detected using a urine dipstick or standard urinalysis [18]; and (5) active malignancy. The institutional review board on research ethics at Nihonkai General Hospital approved the present retrospective cohort study (approval no. 27-1-1). The board waived the requirement for informed consent, because the anonymity of the subjects was insured.

### Definitive diagnosis and assessment of hydronephrosis

As for pain complaints, definitive diagnosis was made using a CT or plain abdominal X-ray of the kidneys, ureters, and bladder (KUB). Helical NCCT or contrast-enhanced CT was conducted at the discretion of emergency physicians. KUB was performed in all patients who did not undergo CT examination in the ED. After the initial presentation, every patient with ureteral stones was on medical follow-up in the urology clinic, until a stone-free status was confirmed. Hydronephrosis was evaluated according to ultrasonography findings performed by a technician or CT performed by an internal radiologist. In cases where both of the modalities were indicated to evaluate hydronephrosis, ultrasonographic data were preferably adopted to maximize the non-radiologic contribution to the present study.

### Meteorological data

Climate information on Sakata city was obtained from an open access database that was publicly provided on the internet by the Japan Meteorological Agency in the Ministry of Land, Infrastructure, Transport, and Tourism (http://www.jma.go.jp/jma/index.html).

### Statistical analysis

We performed univariate analysis using the Chi-squared test or Fisher’s exact test for categorical variables. We used a multivariate logistic regression model yielding the odds ratio (OR), *P* values, and 95 % confidence intervals to identify independent predictors of ureteral stones. This model included variables showing a univariate association (*P* < 0.05) with ureterolithiasis. We analyzed the predictive performance of the multivariate logistic model at an optimal cut-off value determined by calculating the receiver operating characteristic (ROC) curve and area under the curves (AUC). We also calculated sensitivity, specificity, positive predictive value, and negative predictive value at the optimal cut-off value.

*P* < 0.05 was considered statistically significant. All analyses were performed using the R statistical software version 3.1.0 (http://cran.r-project.org/).

## Results

We met a total of 106,146 patients in the institutional ED database between April 1, 2007 and March 31, 2015. Of the patients, 5387 presented with complaints of back, flank, or lower abdominal pain, and 4896 of the 5387 patients were excluded according to the sampling criteria mentioned in the “Patients and methods” section. Specifically, 4426 patients were excluded for insufficient examination, 448 for abnormal signs, and 22 for elevated CRP or active malignancy. Finally, we selected 491 cases who received a definitive radiological diagnosis and did not meet the exclusion criteria. Among the 491 patients selected in the present study, 358 and 133 cases were found to be with or without ureteral stones, respectively (Table 1). The mean age and standard deviation was 49.8 ± 14.2 (range 15–89 years) and 57.3 ± 17.1 (range 18–90 years) for the ureteral stone and non-ureteral stone patients, respectively.

**Table 1:** Comparison of clinical features of patients with and without ureterolithiasis

Factors	Ureteral stone	No ureteral stone	Univariate analysis
	*n* (%)	*n* (%)	*P* value
Age			
<60 years	272 (76.0)	70 (52.6)	1.01*E*−6^†^
≥60 years	86 (24.0)	63 (47.4)	
Sex			
Male	263 (73.5)	83 (62.4)	8.13*E*−6^†^
Female	95 (26.5)	50 (37.6)	
Pain location			
Back	134 (37.4)	57 (42.9)	0.32^†^
None	224 (62.6)	76 (57.1)	
Flank	171 (47.8)	36 (27.1)	5.71*E*−5^†^
None	187 (52.2)	97 (72.9)	
Lower abdomen	136 (38.0)	66 (49.6)	0.026^†^
None	222 (62.0)	67 (50.4)	
Pain onset			
Sudden	211 (58.9)	61 (45.9)	0.013^†^
Not sudden	147 (41.1)	72 (54.1)	
Duration of pain			
<6 h	323 (90.2)	46 (34.6)	<2.2*E*−16^†^
≥6 h	35 (9.8)	87 (65.4)	
Gastrointestinal symptoms			
Nausea or vomiting	111 (31.0)	24 (18.0)	0.0061^†^
None	247 (69.0)	109 (82.0)	
Clinical examination			
Hydronephrosis	266 (74.3)	5 (3.8)	<2.2*E*−16^††^
None	92 (25.7)	128 (96.2)	
Gross hematuria or occult blood in urine	323 (90.2)	29 (21.8)	<2.2*E*−16^†^
None	35 (9.8)	104 (78.2)	
Medical history			
Ureteral stone	98 (27.4)	11 (8.3)	1.06*E*−5^†^
None	260 (72.6)	122 (91.7)	
Hypertension or dyslipidemia	49 (13.7)	32 (24.1)	0.0089^†^
None	309 (86.3)	101 (75.9)	
Diabetes mellitus	16 (4.5)	12 (9.0)	0.086^†^
None	342 (95.5)	121 (91.0)	
Transport to ED			
Ambulance	79 (22.1)	19 (14.3)	0.073^†^
None	279 (77.9)	114 (85.7)	
Arrival time in ED			
1:00 AM–8:30 AM	153 (42.7)	33 (24.8)	0.00041^†^
8:30 AM–1:00 AM	205 (57.3)	100 (75.2)	
Season at presentation			
Hot season^a^	140 (39.1)	26 (19.5)	7.38*E*−5^†^
Other seasons	218 (60.9)	107 (80.5)	

Data are presented as number of patients with and without each factor unless indicated otherwise

*ED* emergency department, *E* exponential

^†^Pearson’s Chi-squared test, ^††^ Fisher’s exact test

^a^Hot season corresponds to summer (July–September) in Japan

**Fig. 1:**
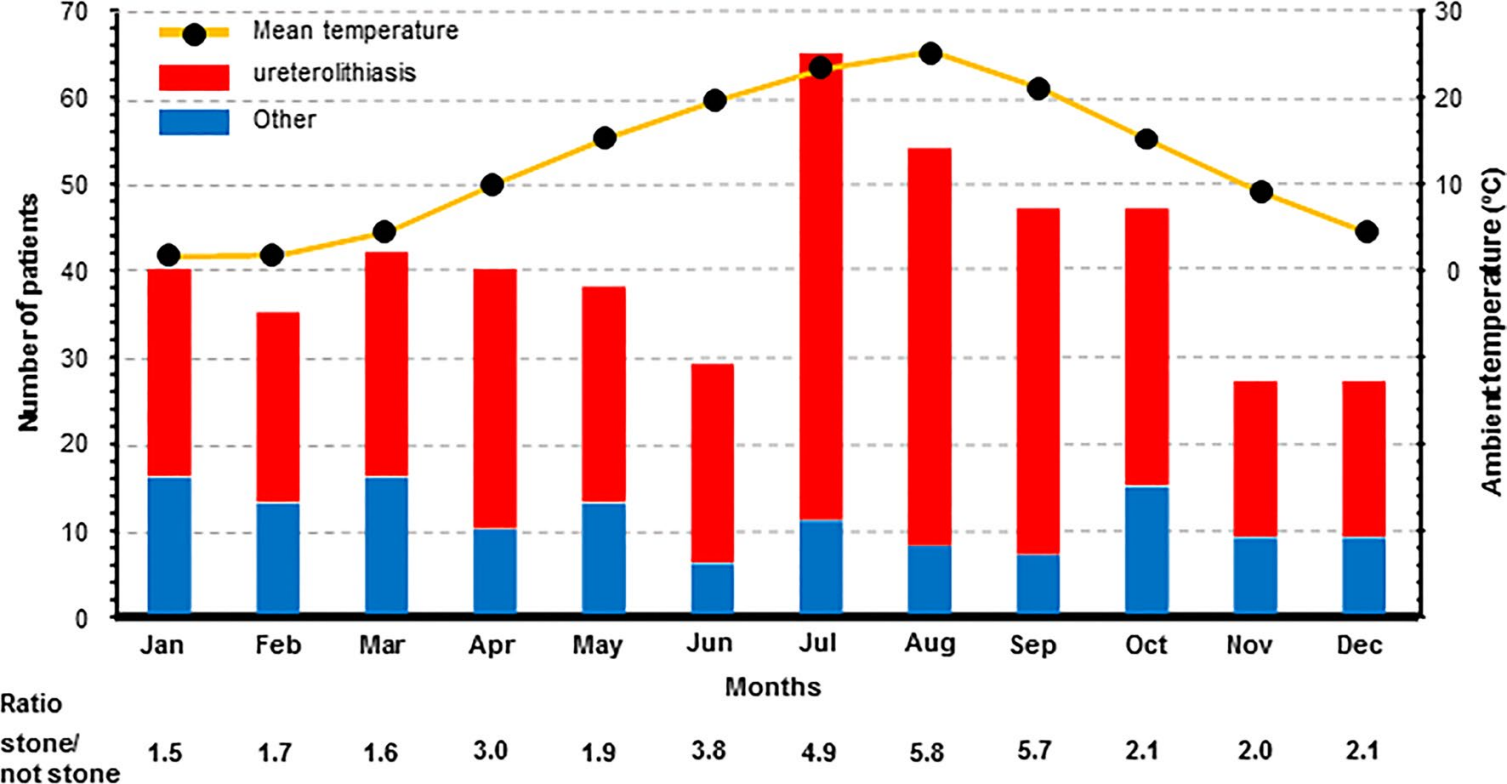
Number of patients with back, flank, or lower abdominal pain visiting the emergency department per month from 2007 to 2015, and the mean ambient temperature in Sakata, Japan, throughout the year. The incidence of ureteral stones varied in parallel with ambient temperature and peaked during the hot season. The number of non-ureteral stone patients did not vary in the same manner, but remained relatively constant throughout the year

As shown in Fig. 1, the number of patients with ureterolithiasis varied depending on the ambient temperature, with the maximum incidence observed during the summer season, July to September. August, which is the hottest month in our region, had the highest ratio of ureteral stone to non-stone diagnoses, at 5.8. In contrast, non-ureteral stone patients having colic mimicking kidney stone attacks visited ED at an almost constant rate throughout the year. Table 1 presents the factors associated with ureterolithiasis determined using the univariate analysis. Compared to each of the references, the following 13 factors were found to be significantly associated with ureteral stones: age, male sex, flank, and lower abdominal pain, sudden onset of pain, short duration of pain (<6 h), nausea or vomiting, hydronephrosis, gross hematuria or occult blood in urine, past history of ureteral stone, co-morbidities of hypertension or dyslipidemia, time at presentation to the ED (1:00 AM–8:30 AM), and summer season (July to September in Japan).

With multivariate logistic regression, eight independent variables associated with the presence of ureteral stone were found to be statistically significant (Table 2): age <60 years, male sex, duration of pain less than 6 h, nausea or vomiting, hydronephrosis, hematuria, past history of ureteral stones, and summer. The remaining items, which were judged as statistically significant with the univariate tests in Table 1, were not found to be significant in multivariate logistic analyses. The results indicate that the summer season is an independent factor for the presence of ureterolithiasis in patients presenting to the ED. Logistic regression demonstrated that the eight factors could be used as significant discriminators between ureteral stone and non-ureteral stone patients. Presentation with back, flank, or lower abdominal pain during the hot season was nine times more likely to be associated with a ureteral stone attack than during other seasons. Presence of hydronephrosis and hematuria raised the likelihood of ureterolithiasis by 46.6 and 39.1 times, respectively, compared with their absence.

**Table 2:** Results of multivariate logistic regression analysis

Factors	Multivariate logistic regression analysis			
	Odds ratio (95 % CI)	Coefficients	SE	*P* value
Age				
<60 years	3.04 (1.20–7.69)	1.1112	0.4735	0.0189
≥60 years	1.00 (Ref.)			
Sex				
Male	3.86 (1.49–9.98)	1.3508	0.4848	0.0053
Female	1.00 (Ref.)			
Duration of pain				
<6 h	10.20 (4.13–25.40)	2.3261	0.4634	5.2*E*−7
≥6 h	1.00 (Ref.)			
Gastrointestinal symptoms				
Nausea or vomiting	3.26 (1.09–9.73)	1.1821	0.5578	0.03410
None	1.00 (Ref.)			
Clinical examination				
Hydronephrosis	46.60 (14.60–149.00)	3.8423	0.5934	9.5*E*−11
None	1.00 (Ref.)			
Gross hematuria or occult blood in urine	39.10 (14.30–107.00)	3.6662	0.5133	9.2*E*−13
None	1.00 (Ref.)			
Medical history				
Ureteral stone	4.42 (1.32–14.80)	1.4855	0.6158	0.016
None	1.00 (Ref.)			
Season at presentation				
Hot season^a^	9.02 (3.13–26.00)	2.1993	0.5395	0.000046
Other seasons	1.00 (Ref.)			

*SE* standard error, *CI* confidence interval, *Ref.* reference value, *E* exponential

^a^Hot season corresponds to summer (July–September) in Japan

The equation for the multiple logistic regression is:

$$\begin{aligned} Z = & - 6.6225 + 1.1112 \times X_{1} + 1.3508 \times X_{2} + 2.3261 \times X_{3} + 1.1821 \\ & \quad \times X_{4} + 3.8423 \times X_{5} + 3.6662 \times X_{6} + 1.4855 \times X_{7} + 2.1993 \times X_{8} , \\ \end{aligned}$$

where *Z* is the linear combination of the variables *X*_1_ to *X*_8_ of the model. Values and explanations of *X*_1_ to *X*_8_ are described in Table 3. The probability of a patient suffering from a stone attack is estimated to be 1/(1 + e^−*Z*^), where e is exponential.

**Table 3:** Variable values in the multiple regression model

Variables	Value	Explanation
*X*_1_	1	Patient aged <60 years
	0	Patient aged ≥60 years
*X*_2_	1	Male
	0	Female
*X*_3_	1	Pain duration <6 h
	0	Pain duration ≥6 h
*X*_4_	1	Presence of nausea or vomiting
	0	None
*X*_5_	1	Presence of hydronephrosis
	0	None
*X*_6_	1	Gross hematuria or occult blood in urine
	0	None
*X*_7_	1	Past history of ureteral stone
	0	None
*X*_8_	1	Hot season^a^
	0	Other seasons

^a^Hot season corresponds to summer (July–September) in Japan

**Fig. 2:**
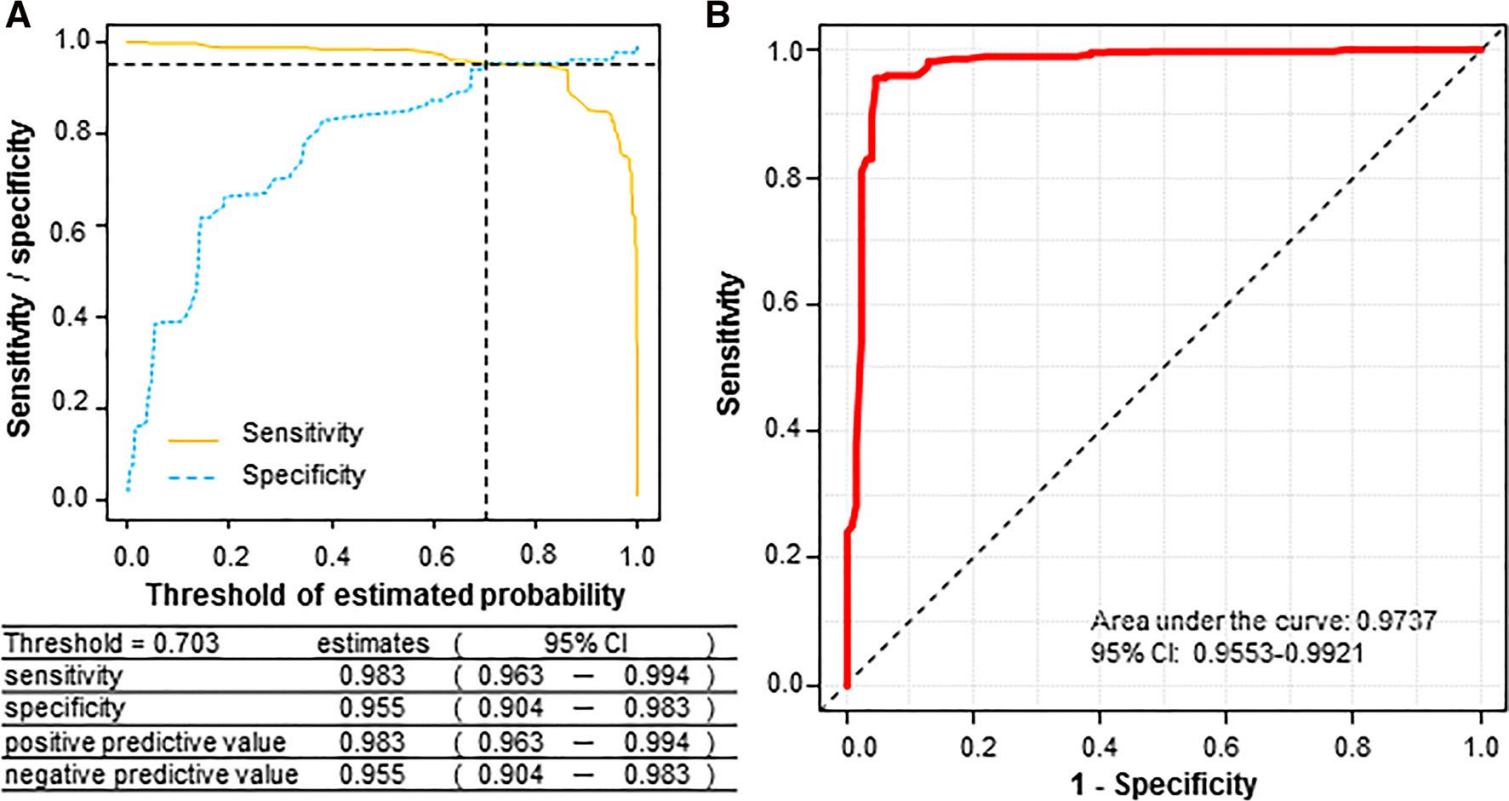
Diagnostic performance of the logistic model. The optimal cut-off value of estimated probability for ureteral stone was set at 0.703 by calculating the intersection of the sensitivity and specificity curves (**a**). The threshold value provides excellent prediction of ureterolithiasis (**b**). Positive and negative predictive values are shown (**a**, *lower*). *CI* confidence interval

**Fig. 3:**
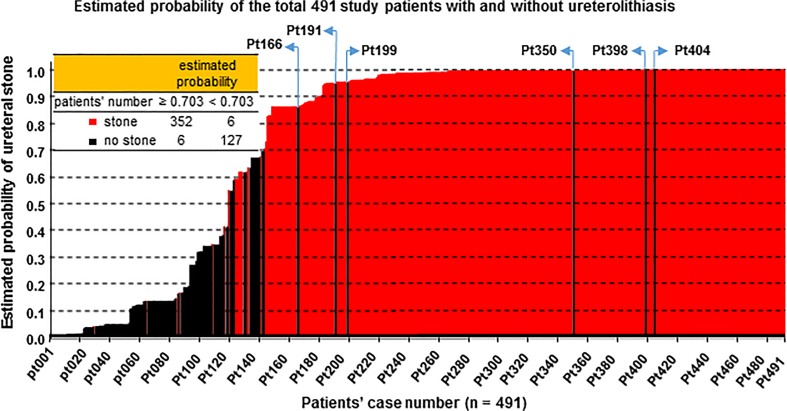
Estimated probability for all 491 patients with and without ureterolithiasis. Of all the 491 study patients, 6 (1.67 %) of 358 with a probability of 0.703 or greater were not found to have ureterolithiasis. See more information on patients with non-ureteral stones in Table 4

Figure 2 shows the diagnostic performance of the logistic model. The optimal threshold value of estimated probability for ureteral stones was calculated to be 0.703 by the intersection of the sensitivity and specificity curves (Fig. 2a), yielding a good prediction for ureteral stones (AUC = 0.9737 in Fig. 2b). It should be noted that the model would have excellent positive and negative predictive values at the optimal cut-off threshold of 0.703 (Fig. 2a, lower). As shown in Fig. 3, however, the model has limitations in interpretation. Of the 491 total study patients, 6 (1.67 %) of the 358 cases with an estimated probability greater than 0.703 were non-stone patients, as detailed in Table 4.

**Table 4:** Differential diagnosis of non-stone patients with estimated probability over 0.703

Case no.	Main causative diseases	Accompanying conditions
pt166	Acute aortic dissection	Asymptomatic ureteral stone in the proximal ureter, solitary and 10 mm in diameter
pt191	Ileus	
pt199	Acute bleeding of renal cyst	
pt350	Retroperitoneal fibrosis	Ureteral stenosis and gastric cancer
pt398	Colonic diverticulitis	
pt404	Retroperitoneal tumor (malignant peripheral nerve sheath tumor)	Ureteral compression

Case no. corresponds to patients’ number at the horizontal axis in Fig. 3

## Discussion

A close relationship between seasons and the incidence of ureterolithiasis has been demonstrated in various areas of the world, including Japan [19], Taiwan [15], the United States [3, 16], Australia [20], New Zealand [6], Italy [21], and Iran [17], where the four seasons rotate in a year, and the temperature fluctuates widely in between the seasons. Our results regarding the relationship between stone-related morbidity and season are consistent with those of previous reports [6, 15, 16, 20, 21]. In many countries, seasonal trends in monthly urinary stone attack rates exist, with the incidence peaking in the summer, which corresponds to July–September [15] and January–March [6] in the northern and southern hemispheres, respectively; these trends have been demonstrated to exist regardless of patients’ age, sex [15], and race [6]. Similarly, in western and northern Iran, autumn has the highest incidence of urinary tract stones [17]. However, the effects of the current season on the differential diagnosis of symptomatically suspected ureteral stones remains poorly understood. In the present study, we demonstrated that seasonal variations were significantly related to ureterolithiasis using a multivariate logistic regression analysis.

An association between temperature and the seasonal pattern of urinary stone disease has been observed [22]. As shown in the present study, numerous studies support the idea of an increased frequency of renal colic with increasing temperature [8, 21]. Two major theories have been proposed. One is the theory of dehydration [21, 23], and the other is an increase in the synthesis of 1,25-dihydroxyvitamin D_3_ (Vit D) due to sunlight exposure [6]. The mechanism of stone formation due to dehydration is an increase in urinary crystallization and stone formation due to the low volume of urine because of insufficient liquid intake to compensate for sweating in hot climates [24]. Alternatively, increased exposure to sunlight causes increased production of Vit D and increased urinary calcium excretion [6]. Serum levels of Vit D and urinary excretion of calcium and oxalate have been shown to be significantly higher during May–October than November–April [25]. In addition, the serum Vit D level was significantly higher throughout the year in hypercalciuric than normocalciuric stone-formers [25]. A population-based study indicated sex differences between the hormonal and dietary control of urinary calcium excretion [26]. Serum calcium level was positively associated with urinary calcium excretion in women but not men [26]. In contrast, serum Vit D level was associated with urinary calcium excretion in men but not women [26]. Transient variations in lifestyle, occupation, or living environment could lead to changes in ureterolithiasis prevalence [7]. In an analysis of desert military deployments moving between temperate and hotter climates, Evans et al. [27] reported that the estimated duration between renal stone formation and presentation of symptoms was 93 ± 42 days (mean ± standard distribution). On the other hand, studying medical records of ED in Iran, Basiri et al. [23] reported that the occurrence of renal colic peaked during warm seasons but was not higher in the holy month of Ramadan, during which Muslims fast from sunrise to sunset for about 12 h a day.

In addition, humidity could influence the onset of renal colic [21]. The stone incidence peak usually coincides with peak temperatures; however, it appeared earlier than the temperature peak in our study. This result may be related to humidity. Boscolo-Berto et al. [21] reported that the onset of renal colic is associated with temperatures above 27 °C and a relative humidity below 45 %. The relative humidity averaged 37.5 % in July and 39.6 % in August from 2007 to 2014 in our region. Thus, humidity differences may cause a gap between temperature and stone incidence peaks. For an alternative reason, increased mobility in the warm season may indirectly influence the onset of renal colic, because the relationship between exercise and urinary stone passage is described in several small-scale studies [28]. The tendency for increasing incidence of renal colic in parallel with the rise in ambient temperature has been well documented in many countries [6, 15, 19]. The prevalence of kidney stones in the United States has risen over the past 30 years. There is a general expectation that the gradual and long-term increase in ambient temperatures due to global warming via greenhouse gases will induce a corresponding rise in ureterolithiasis-related morbidity [3, 15]. Increasing ambient temperatures may increase urinary stone risk by increasing the urinary excretion of calcium and leading to the supersaturation of calcium oxalate and calcium phosphate in the urine independently of environmental humidity, geographic location, and season of the year [8].

However, the trend of increasing prevalence of urinary stones in hot climates is not necessarily universal. The incidence of urinary stones is quite low in Nigeria [29], but extremely high in the Middle Eastern Gulf States, such as Kuwait, the United Arab Emirates, and Saudi Arabia [30]. In addition, renal colic in older men has been shown to be more heavily influenced by hot weather [16]. In addition, sunlight exposure was shown to have no association with lithogenicity in patients with spinal cord injuries [31]. Indeed, the effect of meteorological parameters on renal colic varies depending on age, sex, race, season, and co-morbidity [31]; however, socio-economic conditions may also play a role [32].

The STONE score, developed by Moor and coworkers [14], is a non-radiology-based system that predicts the presence of ureteral stones in patients who present to the ED with flank or back pain. The score consists of five categories: sex, pain duration, race, nausea, and urinary erythrocytes. The influence of racial differences on ureteral stone incidence would be very small in Japan, because most of the inhabitants in Japan are Asian. In fact, the STONE score indicated that the AUC was 0.925 (sensitivity 88.0 %, specificity 88.7 %, positive predictive value 95.5 %, and negative predictive value 73.3 %) in our study population. Instead of race, we selected past history of ureteral stones, age, presence of hydronephrosis, and summertime onset in our model. Adopted based on high *Z* scores, these four items may contribute significantly to stone diagnosis in clinical practice. Daniels et al. [33] emphasized the importance of hydronephrosis in diagnosing ureteral stones, and we also revealed the diagnostic significance of the summer season in the present study. Ureterolithiasis often recurs in a patient’s lifetime [34], supporting the importance of a previous history of stones.

Age (<60 years) is also an important factor in diagnosing ureteral stones and other diseases. The first-episode stone incidence peak was between ages 50–59 in women and 40–49 in men in 2005 in Japan [35]. Epidemiologically, stone colic generally occurs in the younger generation, mainly among individuals aged <60 years [35]. On the other hand, acute appendicitis, colonic diverticulitis, acute aortic dissections, or abdominal aortic aneurysms, which frequently occurred in our study, gradually increase from age 50 and reach a peak at age 60 or older, except for acute appendicitis [36]. Thus, most diagnoses that mimic ureterolithiasis occur mainly among individuals aged 60 years or older.

Since the calculated value is beyond the optimal cut-off threshold of 0.703 as presented in Fig. 2, the value would indicate an excellent prediction for ureterolithiasis (area under the curve = 0.9737). However, our model could not exclude the six non-stone patients who presented with pain suggestive of renal colic and had a probability greater than 0.703 (Table 4; Fig. 3). Hydronephrosis should be checked with ultrasound as a first-line modality. However, CT examination should be considered unless the presence of urinary stone is evident on plain X-ray or ultrasonographic images.

The present study has other limitations in interpretation. The present research was retrospective in nature, was composed of a relatively small number of patients, and was conducted a single medical center. Our prediction logistic model for renal colic has not been validated elsewhere yet. Further prospective and large-scale studies would be necessary to confirm the present findings.

## Conclusions

The incidence of ureteral stones was associated with changes in ambient temperature and peaked during the hot season in Japan, as reported previously in many other countries. The present study indicates that the consideration of the seasonally variable incidence of urinary stone disease is clinically relevant in facilitating proper diagnosis of patients presenting with symptomatically suspected ureteral stones. The prediction model developed in the present study would be useful for screening for ureterolithiasis, despite a small number of false-positive cases.
